# Individual variation of the SARS‐CoV‐2 receptor ACE2 gene expression and regulation

**DOI:** 10.1111/acel.13168

**Published:** 2020-06-19

**Authors:** Jiawei Chen, Quanlong Jiang, Xian Xia, Kangping Liu, Zhengqing Yu, Wanyu Tao, Wenxuan Gong, Jing‐Dong J. Han

**Affiliations:** ^1^ Peking‐Tsinghua Center for Life Sciences Academy for Advanced Interdisciplinary Studies Center for Quantitative Biology (CQB) Peking University Beijing China; ^2^ CAS Key Laboratory of Computational Biology CAS‐MPG Partner Institute for Computational Biology Shanghai Institute of Nutrition and Health Chinese Academy of Sciences Center for Excellence in Molecular Cell Science Collaborative Innovation Center for Genetics and Developmental Biology Shanghai Institutes for Biological Sciences Chinese Academy of Sciences Shanghai China

**Keywords:** ACE2, COVID‐19, SARS‐CoV2

## Abstract

The COVID‐19 coronavirus is now spreading worldwide. Its pathogen, SARS‐CoV‐2, has been shown to use angiotensin‐converting enzyme 2 (ACE2) as its host cell receptor, same as the severe acute respiratory syndrome coronavirus (SARS‐CoV) in 2003. Epidemiology studies found males although only slightly more likely to be infected than females account for the majority of the severely ill and fatality, which also bias for people older than 60 years or with metabolic and cardiovascular diseases. Here by analyzing GTEx and other public data in 30 tissues across thousands of individuals, we found a significantly higher level in Asian females, an age‐dependent decrease in all ethnic groups, and a highly significant decrease in type II diabetic patients of ACE2 expression. Consistently, the most significant expression quantitative loci (eQTLs) contributing to high ACE2 expression are close to 100% in East Asians, >30% higher than other ethnic groups. A shockingly common enrichment of viral infection pathways was found among ACE2 anti‐expressed genes, and multiple binding sites of virus infection related transcription factors and sex hormone receptors locate at ACE2 regulatory regions. Human and mice data analysis further revealed ACE2 expression is reduced in T2D patients and with inflammatory cytokine treatment and upregulated by estrogen and androgen (both decrease with age). Our findings revealed a negative correlation between ACE2 expression and COVID‐19 fatality at both population and molecular levels. These results will be instrumental when designing potential prevention and treatment strategies for ACE2 binding coronaviruses in general.

## Graphical abstract

This study revealed the negative correlation of high basal ACE2 level with CoVID‐19 severity/fatality at the population level and its anticorrelation with virus infection pathway expression levels, upregulation by sex hormones and suppression by inflammatory cytokine at the molecular level.

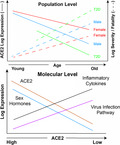

**Abbreviations**
2D: type II diabetes
ACE: angiotensin‐converting enzyme
AGTR: angiotensin II receptor
AngII: angiotensin II
AR: androgen receptor
BMI: body mass index
COVID‐19: coronavirus disease 2019
eQTL: expression quantitative loci
ESR: estrogen receptor
FDR: false discovery rate
GTEx: the genotype‐tissue expression
LVH: left ventricle hypertension
PCC: Pearson correlation coefficients
RAS: renin–angiotensin system
SARS‐CoV‐2: severe acute respiratory syndrome coronavirus‐2
SNP: single‐nucleotide polymorphism
TF: transcription factor
TSS: transcription start site
TTS: transcription termination site
ZNF: zinc‐finger proteins

## 1 INTRODUCTION

Since December 2019, a novel COVID‐19 coronavirus, formally named severe acute respiratory syndrome coronavirus‐2 (SARS‐CoV‐2) (Gorbalenya et al., 2020), exploded in Wuhan, Hubei Province, China, and spread rapidly in China and worldwide. As of March, 25, 2020, 81,285 cases were confirmed in China, and 159 were suspected, with an alarming number of severe cases (CCDC, 2020). Currently, no effective drugs are clinically approved. It is therefore pivotal to find strategies to prevent the virus infection, in particular the severity and fatality associated with it. SARS‐CoV‐2 belongs to the same as the severe acute respiratory syndrome coronavirus (SARS‐CoV) and was shown to use the same receptor, angiotensin‐converting enzyme 2 (ACE2) for entry into the cells through its surface spike (S) protein (Zhou, Yang, et al., 2020). ACE2 plays an important role in regulating the renin–angiotensin system (RAS). Acting as a protease to cleave angiotensin II, ACE2 counteracts the effect of angiotensin II and maintains blood pressure, heart rate, and osmotic pressure (Boehm & Nabel, 2002; Crackower et al., 2002). Despite of not serving as a receptor for other viruses, such as influenza virus H5N1, it also protects mice against both SARS, H5N1 (Zou et al., 2014), H7N9, (Yang et al., 2014) and acid aspiration‐induced lung injury (Imai et al., 2005).

Epidemiology studies have found that males are only slightly more likely than female to be infected, but account for the majority of the severely ill and fatality, and people older than 60 years or have chronic diseases such as type II diabetes (T2D) and hypertension (Chen, Zhou, et al., 2020; Guan et al., 2020; Huang et al., 2020; Wang, Hu, et al., 2020) frequently develop systemic inflammation or cytokine storm, leading to critical conditions of COVID‐19 (Diao, 2020; Huang et al., 2020; Liu, 2020; Wan, 2020). For example, in the analysis of 44,672 confirmed patients in China by February 11, males account for 51.4% of the total patients but 63.8% of the deaths, and the fatalities of males and females are 2.8% compared to 1.7%, respectively (Team & T. N. C. P. E. R. E., 2020). Patients older than 50 years account for nearly half of total confirmed cases, and the majority of fatality is attributed to old males. Globally, World Health Organization has reported 414,179 laboratory‐confirmed cases outside China by March, 25, 2020, mainly distributed in Europe, North America, West Pacific Region, and South‐East Asia, and relatively fewer cases in Africa (WHO, 2020), similar with the distribution pattern of previous SARS‐CoV (WHO, 2003). These suggest that the differences in immunity, gene expression, or even genetic background may contribute to the different susceptibility to and severity of SARS‐CoV‐2 infection. Strangely, in the severe cases, many vital tissues including those with little ACE2 expressed are severely damaged SARS‐CoV‐2 infection (Chai et al., 2020). Since ACE2 as a confirmed host cell receptor for SARS‐CoV‐2 also plays an important role in regulating the renin–angiotensin system (RAS), we examined whether ACE2 expression may contribute to susceptibility and severity of the disease. Here, we found by using the large GTEx data, the association is opposite to that expected from a sole receptive/inductive role of ACE2, with higher ACE2 expression in Asian females, and significantly or insignificantly decreased during aging in many tissues, consistent with the strong ACE2 expression positive eQTLs highly prevalent in East Asians. In addition, using publicly available gene expression datasets, we found ACE2 expression upregulated by estrogen and androgen, downregulated in type II diabetes (T2D) and by inflammatory cytokines in various human and mouse tissues, and a significant negative correlation of ACE2 expression with viral infection, in particular herpes simplex virus 1 infection response genes in many tissues across different individuals.

## 2 RESULTS

### 2.1 ACE2 expression in different tissues across human individuals

**Figure 1:**
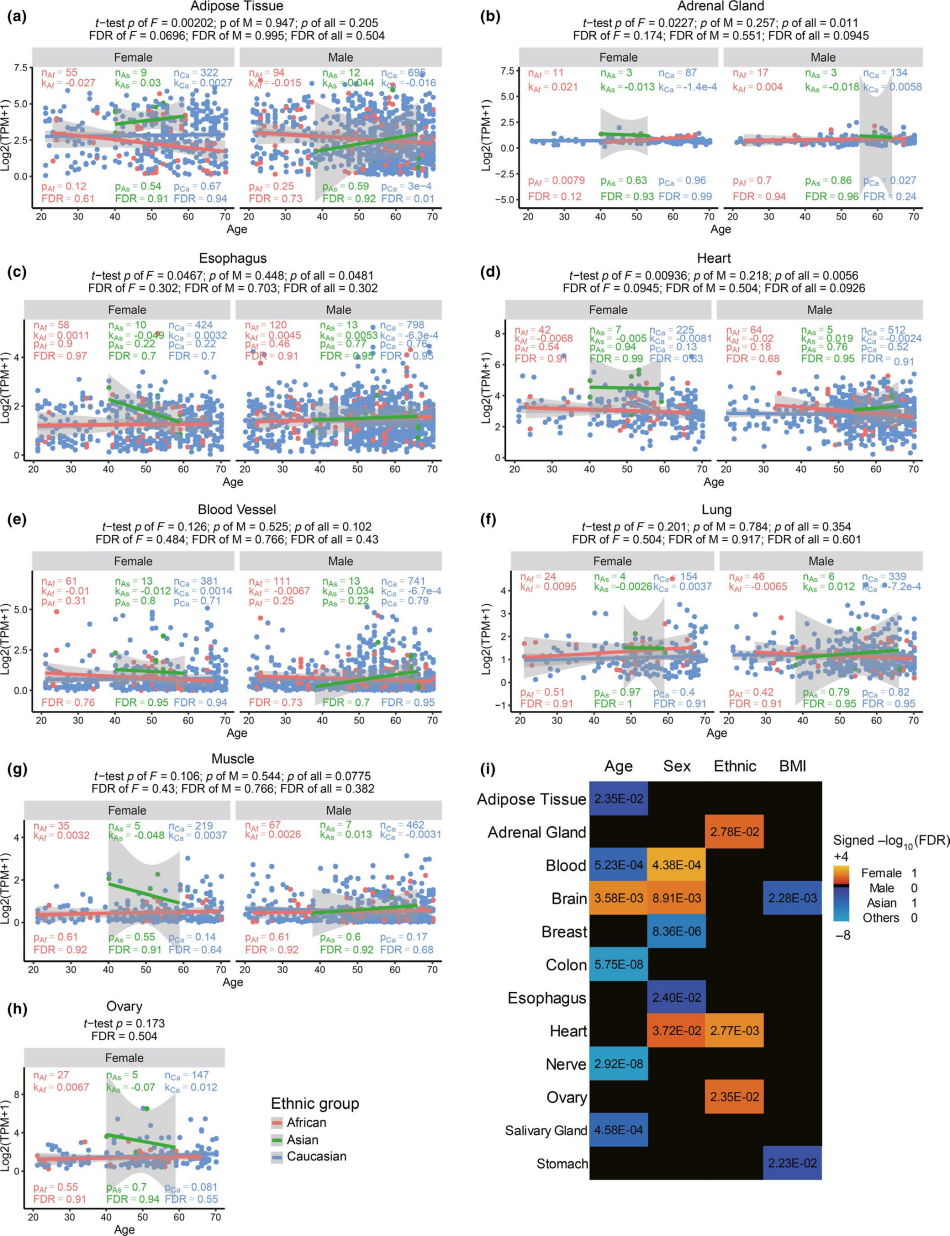
ACE2 expression in different tissues in males, females of Asians, Africans, and Caucasians with age. Panels a–d and e–h are tissues where Asian females show significantly and moderately (marginally significant) higher expression than other groups, respectively. n, sample numbers in each group; k, p, and FDR are slope, *p* value and multiple testing corrected FDR of linear regression, respectively. The significance *p* values and FDR of the difference between Asians versus other ethnic groups are shown on top of the graphs. j, Summary statistics of association with each variable while controlling for other variables

Because both SARS and COVID‐19 preferentially inflict old males (Huang et al., 2020; Karlberg, Chong, & Lai, 2004; Team, 2020) and initially exploded in East Asia, we examined whether ACE2 expression levels are different between male and female, and among the three major ethnic groups. If its sole function is the virus receptor, we would expect old males have the highest level of ACE2, on the contrary, we found among the tissues with large sample sizes and high ACE2 expression, Asian females have significantly higher ACE2 expression in adipose tissue, adrenal gland, heart and esophagus than males, and compared to other ethnic groups (Figure 1a–d), and moderately higher ACE2 expression in blood vessels, lung, muscle, and ovary (Figure 1e–h) (Methods). Although the trend is obvious, after multiple test correction, only adipose tissue reaches an FDR < 0.1. In all ethnic groups, and for both sexes, ACE2 expression generally decreases with age significantly or insignificantly (Figure 1 and Figure S1, Methods). Although only Caucasian samples are large enough to detect significance, it is obvious that the age‐dependent decrease is more significant in males than females. For examples, ACE2 expression significantly decrease with age in colon, blood and adrenal gland, brain, nerve, adipose, and salivary gland in Caucasian males, but only does so in the first three tissues in female (Figure 1 and Figure S1). For colon, nerve, salivary gland, adipose, blood, and brain, even after multiple test correction, the age‐dependent decrease in Caucasian males is significant, but only remains significant for colon in females (FDRs in Figure 1 and Figure S1). These patterns are opposite to an inductive effect of high ACE2 expression to virus infection susceptibility and severity.

The summary statistics show that there is significant association of age, sex, ethnic groups, and body mass index (BMI) with ACE2 expression in many tissues when controlling for rest of the four variables (Figure 1i). The association with age is the strongest, followed by sex, then ethnic groups (Asian versus others), and the BMI, which is negatively associated with ACE2 expression in brain and stomach (Figure 1i).

### 2.2 Complex gene regulatory elements of human ACE2

Next, we wondered why Asian females have higher ACE2 expression than males, and what makes the ACE2 level decrease with age. The human ACE2 gene is mainly composed of two isoforms of 18 or 19 exons, with the longer isoform containing an extra exon at the 5’ end. The coronavirus S protein is known to bind to ACE2 at regions encoded in exons 1, 2, and 8 of the most studied 18‐exon isoform. Here, we refer all annotations according to this 18‐exon isoform.

**Figure 2:**
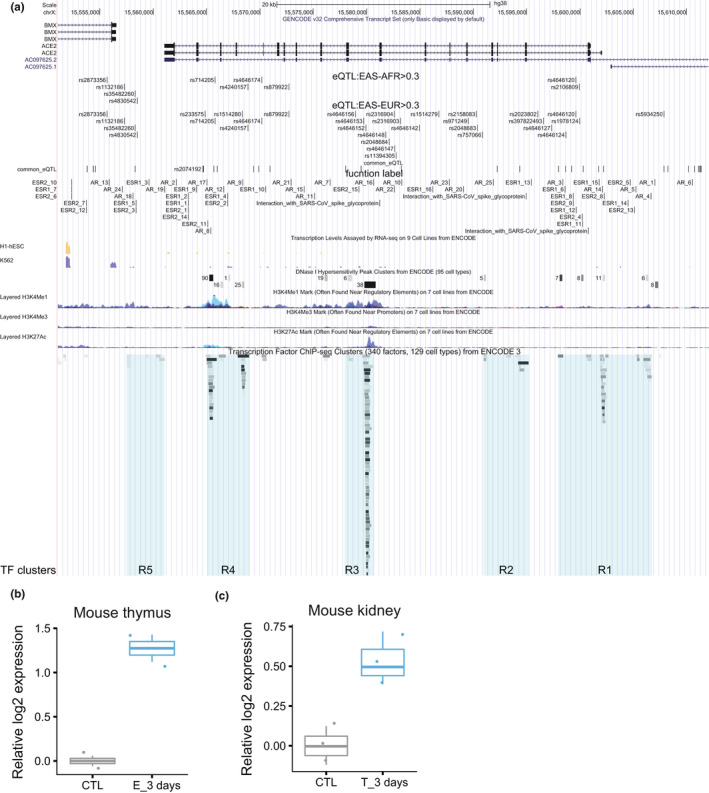
Transcription regulation of human ACE2 gene. (a) Cis regulatory elements. eQTL to ACE2 gene expression in the ACE2 TSS‐10kb to TTS + 10 kb region are visualized together with chromatin modification ChIP‐seq signals, DNAse I sensitivity sites and TF and chromatin state regulators ChIP‐seq targets from ENCODE and ESR1, ESR2, and AR motifs identified by the FIMO function from MEME package. (b,c), The effect of estrogen (b) and testosterone (c) on ACE2 expression in mouse thymus and kidney, respectively

We first analyzed chromatin modification and transcription factor (TF) binding ChIP‐seq profiles to identify key regulatory regions in the −10 kb of transcription start site (TSS) to +10 kb of transcription termination site (TTS) of the ACE2 gene. In addition to the promoter region marked by H3K4me3 and DNAse I hypersensitivity (regulatory region R2), we also identified four enhancer‐like sites marked by H3K4me1, H3K27ac, DNAse I hypersensitivity or TF binding clusters upstream of TSS (R1), downstream of TTS (R5), between exons 8 and 9 (R3) (which is the strongest and most ubiquitous among different cell types), and between 16 and 17 (R4), respectively (Figure 2a) (Methods).

We then calculated ACE2 eQTLs from the GTEx data for each tissue in the TSS‐10kb to TTS+10 kb region of the ACE2 gene (Methods). Although eQTLs are calculated independently, they form 5 clusters, remarkably corresponding to the 5 regulatory regions. In particular, two previously reported SNPs (rs2074192 and rs2106809) that are associated with left ventricle hypertension (LVH) (Fan et al., 2019) fall into R1 at the promoter and the R4, respectively.

All the most significant eQTLs in many tissues, including brain, nerve, artery, ovary, adipose, and liver, have specifically high (close to 100%) allele frequency in East Asians and are all positively correlated with higher ACE2 expression (Table 1). These are apparently consistent with the significantly higher ACE2 expression of East Asian females compared to other ethnic groups independent of age (Figure 1).

**Table 1:** Top 38 most significant eQTLs in ACE2 TSS‐10kb to TTS + 10kb contributing to ACE2 expression

Pos	SNP	Ref	Alt	eqtl(pvalue,NES,tissue)	EAS‐EUR	EAS‐AFR	AMR_AF	AFR_AF	EUR_AF	SAS_AF	EAS_AF
15,599,940	rs1978124	T	C	9.5e−17,0.28,Nerve ‐ Tibial	0.465	0.093	0.710	0.900	0.529	0.781	0.994
15,607,860	rs5934250	T	G	1.5e−16,0.28,Nerve ‐ Tibial	0.462	0.093	0.710	0.900	0.531	0.780	0.994
15,599,613	rs4646120	G	A	2.9e−14,0.25,Nerve ‐ Tibial	0.466	0.305	0.689	0.689	0.527	0.781	0.994
15,581,769	rs4646148	T	TTTAA	2.5e−09,0.21,Nerve ‐ Tibial	0.341	0.101	0.758	0.892	0.653	0.802	0.994
15,594,320	rs397686765	GA	G	3.3e−09,0.2,Nerve ‐ Tibial	‐	‐	‐	‐	‐	‐	‐
15,596,022	rs2023802	G	A	4e−09,0.2,Nerve ‐ Tibial	0.342	0.188	0.752	0.806	0.651	0.802	0.994
15,598,673	rs4646124	T	C	4e−09,0.2,Nerve ‐ Tibial	0.342	0.190	0.752	0.804	0.651	0.802	0.994
15,578,920	rs4646156	A	T	4e−09,0.2,Nerve ‐ Tibial	0.342	0.190	0.752	0.804	0.651	0.797	0.994
15,596,540	rs397822493	G	GT	4.5e−09,0.21,Nerve ‐ Tibial	0.341	0.077	0.765	0.916	0.653	0.802	0.994
15,578,020	rs4646158	C	CATAAG	4.5e−09,0.21,Nerve ‐ Tibial	‐	‐	‐	‐	‐	‐	‐
15,597,330	rs4646127	A	G	7.5e−09,0.2,Nerve ‐ Tibial	0.342	0.170	0.754	0.824	0.651	0.802	0.994
15,597,980	rs60097061	C	CA	7.5e−09,0.2,Nerve ‐ Tibial	‐	‐	‐	‐	‐	‐	‐
15,590,376	rs2048683	T	G	8.5e−09,0.2,Nerve ‐ Tibial	0.346	0.187	0.750	0.807	0.648	0.802	0.994
15,582,756	rs11394305	C	CA	8.6e−09,0.2,Nerve ‐ Tibial	0.337	0.096	0.75,0	0.889,0.002	0.6490,0	0.800,0	0.986,0.003
15,582,568	rs2316904	C	T	8.6e−09,0.2,Nerve ‐ Tibial	0.343	0.101	0.756	0.892	0.650	0.802	0.994
15,582,621	rs4646147	T	A	8.6e−09,0.2,Nerve ‐ Tibial	0.342	0.101	0.756	0.892	0.651	0.804	0.994
15,583,151	rs2316903	G	T	8.6e−09,0.2,Nerve ‐ Tibial	0.342	0.101	0.756	0.892	0.651	0.802	0.994
15,589,527	rs971249	T	C	1e−08,0.2,Nerve ‐ Tibial	0.346	0.187	0.748	0.807	0.648	0.802	0.994
15,590,829	rs757066	C	T	1.2e−08,0.2,Nerve ‐ Tibial	0.345	0.006	0.782	0.988	0.649	0.802	0.994
15,586,742	rs1514279	G	A	1.3e−08,0.2,Nerve ‐ Tibial	0.347	0.187	0.750	0.807	0.646	0.802	0.994
15,579,712	rs4646153	C	T	1.6e−08,0.2,Nerve ‐ Tibial	0.345	0.084	0.756	0.909	0.649	0.802	0.994
15,579,901	rs4646152	A	G	1.6e−08,0.2,Nerve ‐ Tibial	0.342	0.084	0.756	0.909	0.651	0.802	0.994
15,582,092	rs2048684	A	C	1.6e−08,0.2,Nerve ‐ Tibial	0.342	0.084	0.756	0.909	0.651	0.802	0.994
15,590,263	rs2158083	C	T	1.9e−08,0.19,Nerve ‐ Tibial	0.346	0.170	0.752	0.824	0.648	0.802	0.994
15,599,938	rs2106809	A	G	6.4e−08,0.22,Nerve ‐ Tibial	0.276	0.434	0.324	0.089	0.247	0.483	0.522
15,584,941	rs4646142	G	C	5.5e−07,0.44,Brain ‐ Nucleus accumbens	0.300	0.298	0.336	0.240	0.238	0.479	0.538
15,592,225	rs756737634	C	CT	5.5e−07,0.44,Brain ‐ Nucleus accumbens	‐	‐	‐	‐	‐	‐	‐
15,557,256	rs1132186	A	C	1.6e−06,0.41,Brain ‐ Substantia nigra	0.315	0.488	0.716	0.476	0.649	0.715	0.963
15,558,292	rs35482260	GT	G	2.9e−06,0.33,Brain ‐ Caudate	−0.003	−0.023	0,0.720	0.023,0.473	0.003,0.647	0,0.714	0,0.959
15,564,843	rs233575	G	A	3.1e−06,0.35,Brain ‐ Caudate	0.332	0.008	0.786	0.988	0.665	0.816	0.996
15,570,148	rs4646174	C	G	3.7e−06,0.41,Brain ‐ Substantia nigra	0.315	0.510	0.721	0.454	0.649	0.715	0.963
15,555,645	rs2873356	A	C	4.2e−06,0.41,Brain ‐ Substantia nigra	0.343	0.303	0.748	0.693	0.653	0.801	0.996
15,568,325	rs1514280	A	G	6.2e−06,0.33,Brain ‐ Caudate	0.341	0.205	0.754	0.792	0.655	0.802	0.996
15,565,781	rs714205	C	G	8.1e−06,0.42,Brain ‐ Nucleus accumbens	0.330	0.435	0.303	0.101	0.205	0.471	0.535
15,572,684	rs879922	C	G	1.1e−05,0.3,Brain ‐ Caudate	0.317	0.510	0.720	0.454	0.646	0.715	0.963
15,558,483	rs4830542	C	T	1.7e−05,0.3,Brain ‐ Caudate	0.315	0.506	0.720	0.458	0.649	0.716	0.963
15,568,841	rs4240157	C	T	1.7e−05,0.3,Brain ‐ Caudate	0.322	0.507	0.720	0.457	0.641	0.715	0.963

Note

Yellow highlights the eQTLs with AF difference between EAS and EUR > 0.3, green between EAS and AFR > 0.3.

For each eQTL, only the most significant tissue is shown. A complete list of tissues and all significant eQTLs are shown in Table S1.

It was previously reported that estrogen is protective against SARS infection in mice (Channappanavar et al., 2017). We asked whether ACE2 expression is regulated by estrogen or androgen (testosterone). Interestingly, among these regulatory regions there are many estrogen receptor binding motifs and a few androgen receptor binding motifs. In particular, R1 and R4 contain clusters of multiple ESR binding motifs and more tissue‐specific enhancer marks than the more ubiquitous R3 (Figure 2a).

### 2.3 Regulation of ACE2 expression by estrogen and androgen

To test whether the sex hormones, which decrease with age, can regulate ACE2 expression, we searched the GEO database for RNA‐seq/microarray datasets upon estrogen or androgen treatment. Among these data, we found estrogen treatment significantly increases ACE2 expression by an average of 1.274 log2 fold in mouse thymus (GSE2889, *p* = .005, FDR = 0.028, Figure 2b), whereas androgen (testosterone) increases ACE2 expression in mouse kidney by an average of 0.533 log2 fold (GSE47181, *p* = .017, FDR = 0.137, Figure 2c). Interestingly, while ACE2 expression in high in infants and highest in adolescent and decrease dramatically in adult males, transgender males who underwent estrogen therapy (estradiol) and androgen deprivation therapy (spirolactone) for 1 year show significantly higher ACE2 expression level and more ACE2 expressing cells among testis Sertoli cells (Figure S2). It should be noted, similar to the ACE2 expression decline over age, both estrogen and androgen are well known to decrease with age (Horstman, Dillon, Urban, & Sheffield‐Moore, 2012).

### 2.4 Potential function of ACE2 in antiviral response

**Figure 3:**
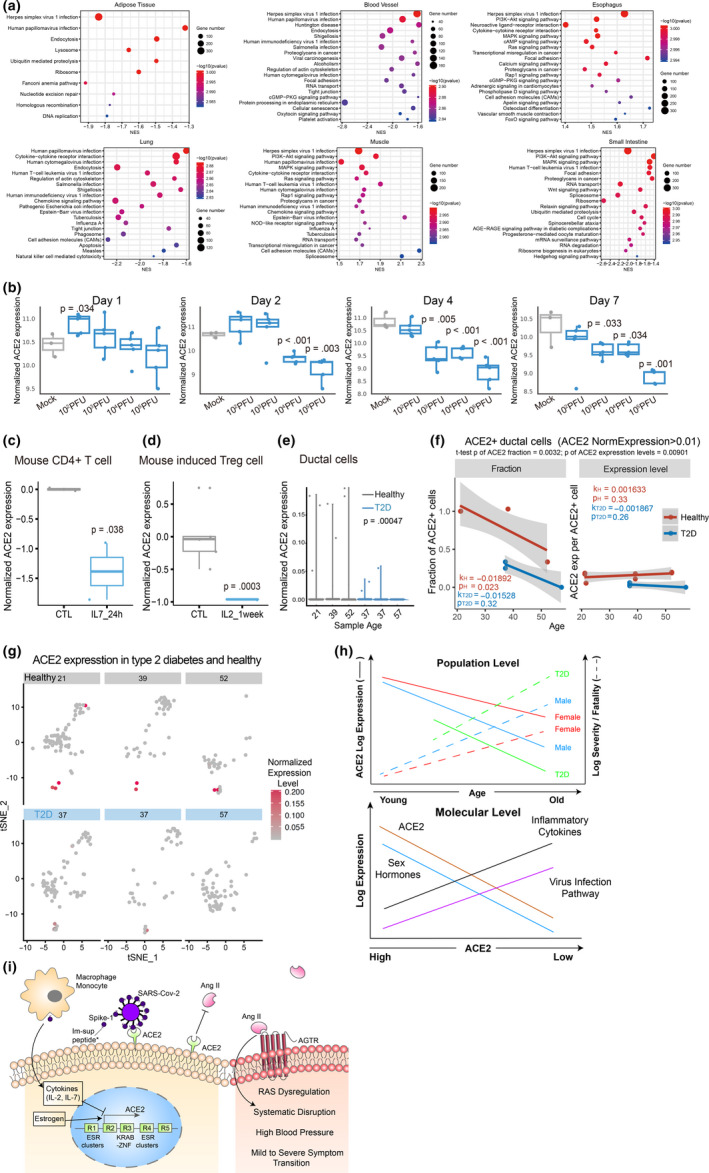
ACE2 expression is negatively with virus infection pathways and downregulation by inflammation cytokines and in T2D. (a) KEGG pathway enrichment of ACE2 correlated and anti‐correlated genes in each tissue were determined by GSEA (Methods). (b) Dosage and time‐dependent SARS‐CoV infection on ACE2 mRNA level. (c–d), Suppression of ACE2 expression by IL2 and IL‐7 treatment in mouse T cells. (e) Reduction of ACE2 expression in T2D human pancreas ductal cells compared to age‐matched controls as shown by scRNA‐seq data. f, Fraction of ACE2 + cells among ductal cells and the average ACE2 expression per ACE2 + ductal cell in healthy and T2D individuals. ACE2 + cells are defined by normalized expression level > 0.01. k and p, slope and *p* value of linear regression to age, respectively. The significance *p* values of the difference between healthy and T2D groups are shown on top of the graphs. (g) ACE2 expression patterns projected on the t‐SNE (t‐distributed stochastic neighbor embedding) plot from type 2 diabetes and healthy pancreas single‐cell RNA‐seq data. (h) Schematic illustrations of the negative correlation of high basal ACE2 level with CoVID‐19 severity/fatality at the population level (top, solid lines indicate our findings; dotted lines are knowledge derived from literature), and its anti‐correlation with virus infection pathway expression levels, upregulation by sex hormones, and suppression by inflammatory cytokine at the molecular level (bottom). (i) A putative model illustrating the potential regulations and functions of the ACE2 gene. *Function of immunosuppressive peptide and their proposed action on macrophage and monocyte are from (Gallaher & Gallaher, 2020; Sarzi‐Puttini et al., 2020)

In the majority of human tissues across different individuals, the top most enriched pathway transcriptionally anti‐correlated with ACE2 is Herpes simplex virus 1 (HSV1) infection, in lung, is human papillomavirus (HPV) infection (Figure 3a, Figure S3). The virus infection‐related genes negatively correlated with ACE2 expression in intestine, breast, and ovary include the APOBEC RNA (often viral RNA) editing enzymes, and most prominently many ZNF (shockingly all are KRAB‐ZNF containing the transcriptionally repressive KRAB domain) transcription factors, which often directly binds to endogenous retrovirus sequences (Table S2). At least 3 KARB‐ZNFs directly bind to the ACE2 R3 region (Figure S4). Intriguingly, these genes are strongly positively correlated ACE2 expression in testis and pancreas (Table S2), but not in pancreas ductal cells, where the most significant KEGG negatively associated with ACE2 expression is again herpes virus infection (Figure S5d‐e). Interestingly, consistent with ACE2's anti‐hypertension function, fatty acid metabolism is the major metabolic pathway associated with ACE2 expression in colon and salivary gland (Figure S3).

Although by directly binding and consuming ACE2, the ACE2 protein expression is expected to decrease by SARS‐CoV infection as reported (Kuba et al., 2005), it is puzzling why virus infection by those that do not use ACE2 as receptor, such HSV1, HIV or HPV show any positive or negative correlation with ACE2 expression. ACE2 protein expression is repressed by lung injury caused by acid inhalation (Imai et al., 2005), H5N1 (Zou et al., 2014), and H7N9 (Huang et al., 2014), and ACE2 KO mice have been shown to be more susceptible to lung injury caused by acid inhalation (Imai et al., 2005), H5N1 (Zou et al., 2014), H7N9 (Yang et al., 2014) and SARS‐CoV (Kuba et al., 2005). Consistently, we found using a previously published microarray dataset on mice lung upon SARS‐CoV infection (Gralinski et al., 2018; Totura et al., 2015), ACE2 mRNA is transiently upregulated at low SARS‐CoV titer but repressed at higher dosage or with time after infection, by day 4 even the lowest dosage tested suppressed ACE2 expression (Figure 3b). The fact that ACE2 seriously decreases in the lung of SARS‐CoV‐infected mice is consistent with the increase of AngII level, leading to vascular permeability and loss of vascular homeostasis (Kuba et al., 2005; Liu, 2020).

Since cytokine storm was shown to underline the severity of the infection (Huang et al., 2020; Wan, 2020), we wonder whether some of the differentially expressed cytokines between mild and severe symptom patients can suppress ACE2 expression. These include the significant increase of IL‐2, IL‐7, IL‐10, G‐CSF, IP‐10, MCP‐1, MIP‐1a, and TNF‐α in severe symptom patients (Huang et al., 2020). We found using public gene expression data, IL‐7 significantly decreased Ace2 expression in mouse CD4 T cells (GSE86542, logFC = −1.386, *p* = .038, FDR = 0.282) and IL‐2 decreased Ace2 expression in induced Treg cells from Foxp3/EGFP bicistronic mice (GSE14415, logFC = −0.962, *p* = .0003, FDR = 0.023) (Figure 3c and d). As T2D is highly associated with COVID‐19 fatality (Yang et al., 2020), we compared ACE2 expression in T2D versus control human subjects. Interestingly, ACE2 expression is significantly decreased in at least in female adipose tissue and male muscle in T2D patients (Figure S5a and b). A single‐cell RNA‐seq data showed ACE2 is expressed in ductal cells of pancreas (Figure 3e–g and Figure S5), and both ACE2 expression and the fraction of ACE2 + cells among all ductal cells and significantly decrease in T2D patients (GSE83139, *p* = .00047 and 0.0032, respectively). The fraction of ACE2 + cells also slightly decreases with age, while the average ACE2 expression per ACE2 + ductal cell is largely unchanged with age, but decreases in T2D (*p* = .00901, Figure 3f).

## 3 DISCUSSION

In summary, through integrating public genomics, epigenomics, and transcriptomics data, we examined whether variation of the SARS‐CoV‐2 receptor ACE2 gene expression in different tissues across individuals can explain the differences in infection susceptibility and outcome. Our findings are contrary to the expectation from ACE2 being only a receptor for the virus, instead, its expression level is high in Asian females and young people (Figure 1 and Table 1), those who are known to be less susceptible, and even less inflicted by severe or fatal outcome, while it is low in males, further decrease with age and T2D, those who are most susceptible to bad outcome (Figures 1 and 3), suggesting at a population level a negative correlation between ACE2 expression and COVID‐19 severity and fatality.

More and more studies have shown COVID‐19 is a systematic disease, and multi‐organ dysfunction is a main characteristic for its severity and mortality (Chen, Wu, et al., 2020; Ruan, Yang, Wang, Jiang, & Song, 2020; Wang, Du, et al., 2020). Researches showed that SARS‐CoV‐2 attack many organs other than the lung, such as cardiovascular organs, directly or indirectly by cytokine storm, which is a major factor driving patients into critical conditions (Inciardi et al., 2020; Moore & June, 2020). Thus, the patterns we found on the ACE2 expression in multiple organs, not limited to lung, among different populations and under different conditions, might offer an explanation of why people in certain population are more susceptible to severe or fatal symptoms than others after the infection. Furthermore, since adipose tissue is a major organ generating inflammatory cytokines (Tchkonia et al., 2010), the fact that we found the most significant age‐dependent and ethnic group dependent ACE2 expression difference and eQTL in this tissue suggest that the ACE2 expression level in this tissue might be associated with cytokine storm seen in severe cases.

At the molecular level, we found ACE2 anti‐expressed genes in most tissues and cells are highly enriched for virus infection pathways (Figure 3a), estrogen strongly and androgen moderately increase ACE2 expression in mouse and human tissues (Figure 2), whereas severe COVID‐19 induced IL‐2 and IL‐7 repress ACE2 expression in mouse T cells, and T2D reduced ACE2 expression and ACE2 expressing cells in human tissues (Figure 3). After being infected with SARS‐CoV‐2, both IL‐7 and IL‐2 in the infected individuals show a significant increase (Huang et al., 2020; Liu, 2020). The number of CD4^+^ T cells and Treg cells in the critically ill patients has a downward trend (Chen, Qi, et al., 2020; Qin et al., 2020; Zhou, Fu, et al., 2020). Our study observed that the ACE2 level in CD4 T cells and Treg cells decreased in mice treated with IL‐7 and IL‐2, respectively, which suggests that the cytokine storm from SARS‐CoV‐2 severe symptom patients may have a decline of ACE2, which in turn further harm CD4^+^ T cells and Treg cells. Consistent with this notion, the autopsy results have shown large‐scale and pervasive damage to organs such as spleen and lymph nodes, where these immune cells reside (Cao, 2020; Shi et al., 2020). The effect of estrogen, androgen and cytokines are reflected on clusters of estrogen and androgen receptors, STAT5, JUN, MYC and other TFs binding at ACE2 regulatory regions, where hypertension QTLs and the great majority of ACE2 eQTLs are also mapped to. Finally, the eQTLs that show largest allele frequency differences between ethnic groups are also the ones having close to full penetrance in East Asians and also the strongest positive eQTLs on ACE2 expression (Table 1). Our results suggest that at least estrogen might contribute the higher ACE2 expression in Asian females than Asian males (Figure 3i), in that estrogen treatment alone or estrogen plus androgen block dramatically induce ACE2 expression (Figure 2b and Figure S2), and the decline of sex hormones can contribute to ACE2 expression decrease with age (Figure 3i), in that both estrogen and androgen can increase ACE2 expression, although estrogen appears to have a stronger inductive effect than androgen (Figure 2b).

Although we have analyzed thousands of samples, the sizes of Asian samples and samples for some tissues are still small compared to others, it remains to be seen whether larger samples will reveal similar patterns in the future when more data become available. It should also be noted that we only investigated the mRNA level changes of ACE2, whether its protein level shows similar changes needs to be further investigated, and many of the ACE2 changes do not occur in lung, the key afflicted tissue in COVID‐19, in fact lung does not have high expression of ACE2 compared to many other tissues. The direct SARS‐CoV‐2 binding of ACE2 protein as the virus receptor might also decrease the ACE2 protein levels. Before the availability of an effective vaccine to prevent SARS‐CoV‐2 infection, a major task is to understand the variations in severity and fatality of the infection in human populations, to which ACE2 might be one of the contributors. Fortunately, the low ACE2 activity can be rescued by dampening its negatively regulated downstream targets such as angiotensin II or its receptors, such as by angiotensin II antagonist losartan (Yan et al., 2015). While this study raises more questions than answers, it is important in that it is a call to action to better understand how COVID‐19 (and other viruses) interact with ACE2 and related inflammation. Answers to these questions may define how therapeutics can be developed.

## 4 METHODS

### 4.1 GTEx gene expression data and analysis

The gene TPM data were obtained from the GTEx Portal and dbGaP accession number phs000424.v8.p2. Metadata (ethnic and sex information) were obtained from phs000424.v8.pht002742.v8.p2.c1.GTEx_Subject_Phenotypes.GRU.txt. For each tissue with more than 20 samples (27 tissues altogether), linear regression analyses and significance tests of ACE2 expression to age in males and females of the three ethnic groups were obtained by lm(Expression ~ Age) and *t*.test(Asian, others) in R. Multiple testing BH corrected FDR of the *t*‐test *p* values and linear regression *p* values were obtained by *p*.adjust(*p* values, method = fdr") in R based on all 27 tested tissues.

To determine the individual effect of age, sex, ethnic, and BMI after controlling for the rest of the four factors on ACE2 expression level, we used the following linear model as in Lehallier et al., 2019):

ACE2level∼α+β1age+β2sex+β3ethnic+β4BMI+ε.

The type II sum of squares was calculated using the ANOVA function of the R car package. This sum of squares type tests for each main effect after the other main effects. *q* values were estimated using the Benjamini–Hochberg approach. The scatter plots were plotted by R package “ggplot2.” Nineteen tissues with more than 10 ACE2‐positive (log2(TPM + 1) ≥ 2) samples were used to calculate expression PCC between each gene and ACE2. In each tissue, the genes ranked by PCC were used to test KEGG pathway enrichment by GSEA.

### 4.2 Calculation of eQTLs of ACE2

SNPs of ±10 kb of *ACE2* were selected from dbSNP build 150. These SNPs were used to calculate eQTLs for all tissues of GTEx by the “eQTL Calculator” from GTEx. eQTLs with *p* value < .01 were retained. Population allele frequencies were obtained from 1,000 Genomes Phase 3.

### 4.3 UCSC genome browser track view of ACE2 gene regulatory regions

The ENCODE track of transcription, histone modification (H3K4me1, H2K4me3, and H3K27ac), and transcription factor ChIP‐seq clusters are selected to show around ACE2 gene (including ± 10 kb of the whole gene). In parallel, we added the track of eQTLs that has large difference between populations, all eQTLs on ACE2, and functional annotation of ACE2 gene (including inferred motif binding region of ESR1, ESR2 and AR, calculated by FIMO from MEME suite with default settings, and inferred interaction sites with SARS‐CoV spike glycoprotein from the UniProt database).

### 4.4 Public data for perturbation effect on Ace2 expression

The GEO database was searched for perturbations, including estrogen, androgen, testosterone, IL‐2, IL‐7, IL‐10, G‐CSF, IP‐10, MCP‐1, MIP‐1a, and TNF‐α according to the significantly differentially expression between mild and severe symptom patients (Huang et al., 2020), on both human and mouse normal tissue or cell lines. Log2 fold change (logFC) relative to controls, p, and FDR (adjusted p value by BH correction) was calculated by using the GEO2R R script and visualized. For platform have several probes or several experimental setups for Ace2, the one with smallest p is shown. Mouse lung SARS‐CoV infection microarray was downloaded from the GEO database (GSE33266), and log2 expression levels were visualized.

### 4.5 Analysis of ACE2 expression in ductal cells

GEO dataset (GSE83139) was used for analysis. The Seurat program (https://satijalab.org/seurat/, v.3.1.4) was used to read a combined gene‐barcode matrix of all samples. Each sample was filtered and normalized with default settings. Specifically, cells were retained only if they contained >200 expressed genes and had <25% reads mapped to mitochondrial genome. t‐SNE and clustering analyses were performed on the combined data using the top 2,500 highly variable genes and 1–15 PCs, which showed the most significant *p* values. The expression level of highly variable genes in the cells was scaled and centered for each gene, then used for t‐SNE plot.

### 4.6 Analysis of ACE2 expression in human testis

GEO datasets (GSE134144, GSE112013) were used for bioinformatics analysis. We used Sctransform (https://satijalab.org/seurat/v3.0/sctransform_vignette.html,v0.2.1) to merge, normalize, and stabilize the technical noise variance of UMI counts prior of adult, adolescent, and transgender samples’ data. Each experiment was filtered and normalized with default settings. Specifically, cells were retained only if they contained >500 expressed genes and had <25% reads mapped to mitochondrial genome. A total of 19,240 testis cells were retained in for downstream analysis. Dimensionality reduction by Seurat and Monocle (https://cole‐trapnell‐lab.github.io/monocle3/,v2.14.0) was used for visualization and further exploration of results.

## CONFLICT OF INTEREST

The authors declare no conflict of interests.

## AUTHOR CONTRIBUTIONS

J.D.J.H. conceived the project, instructed the analyses, and wrote the paper with help from others. All others implemented the analyses.

## Supporting information

Fig S1‐S5

Tab S1‐S3

## Data Availability

All data analyzed in this study are publicly available.
