# Supplementary material for: Individual variation of the SARS‐CoV‐2 receptor ACE2 gene expression and regulation
Source: Aging Cell. 2020 Jun 19;19(7):e13168. doi: 10.1111/acel.13168 (PMC7323071; doi:10.1111/acel.13168)

Supplementary Figure 1

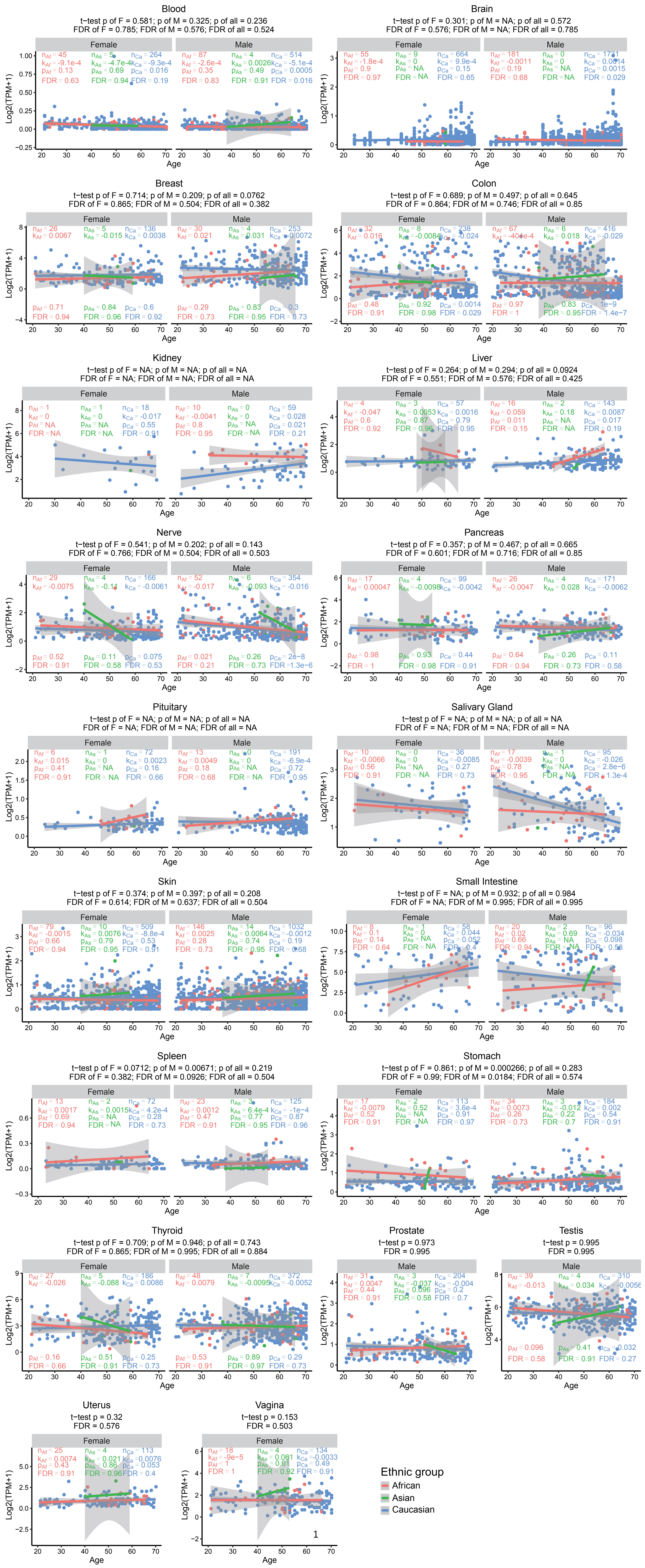

Supplementary Figure 2

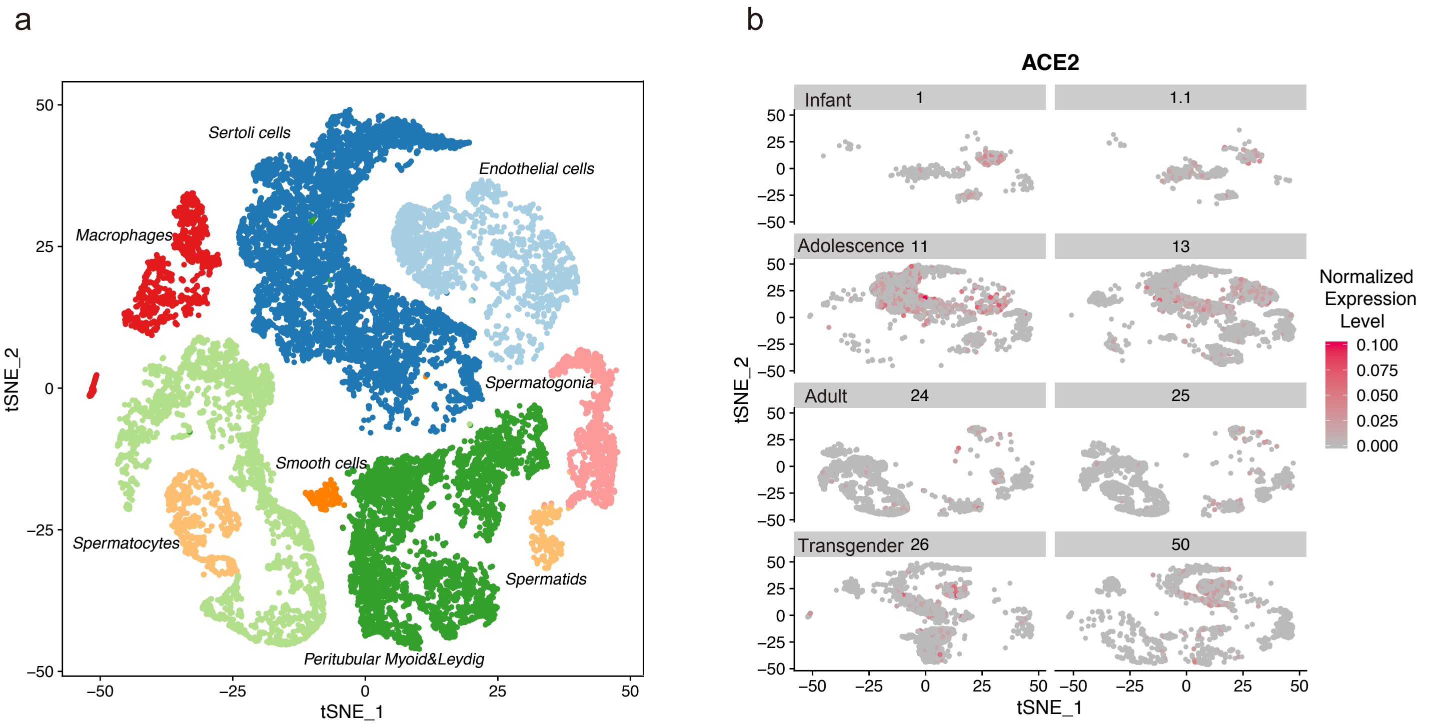

Supplementary Figure 3

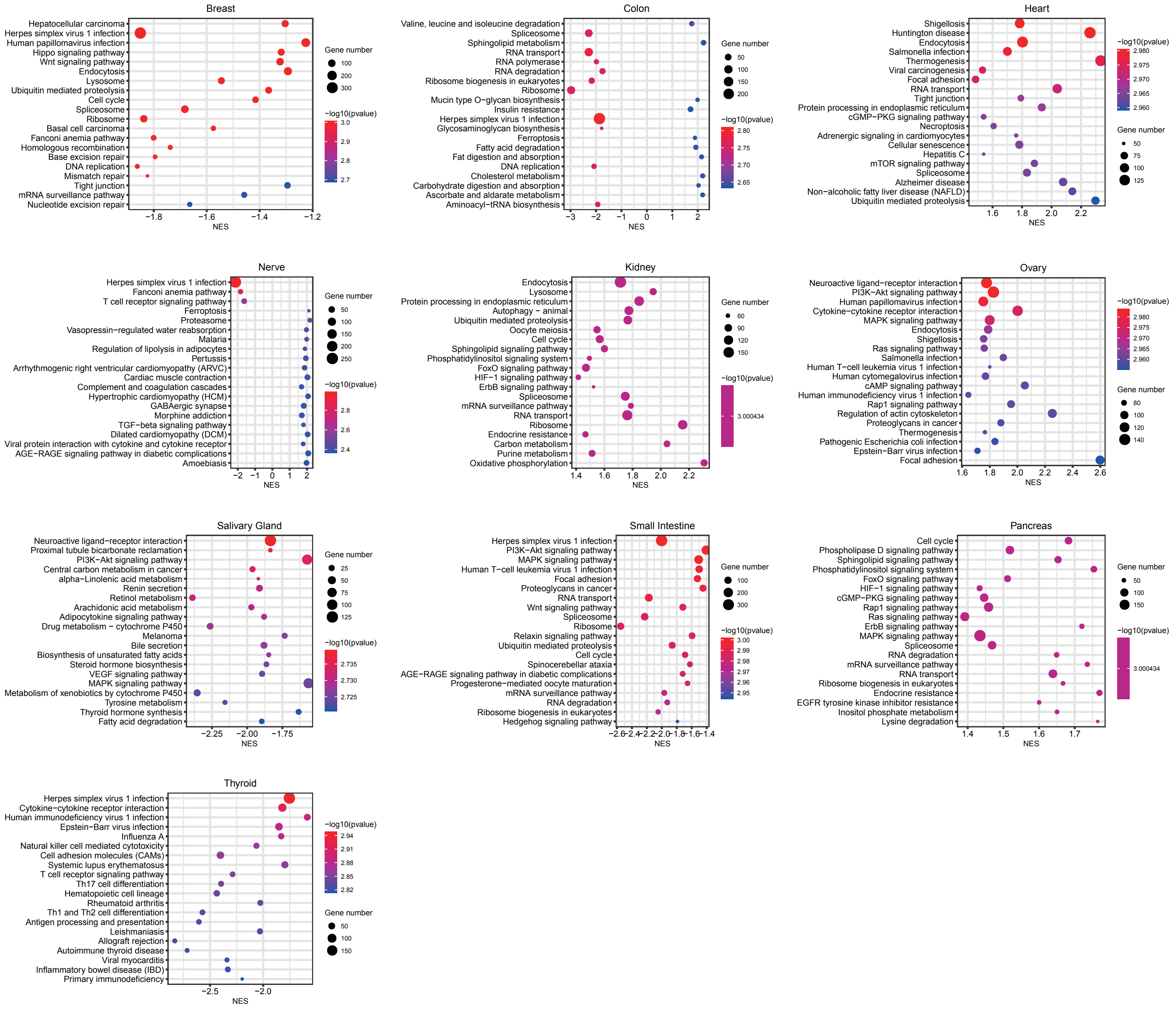

Supplementary Figure 4

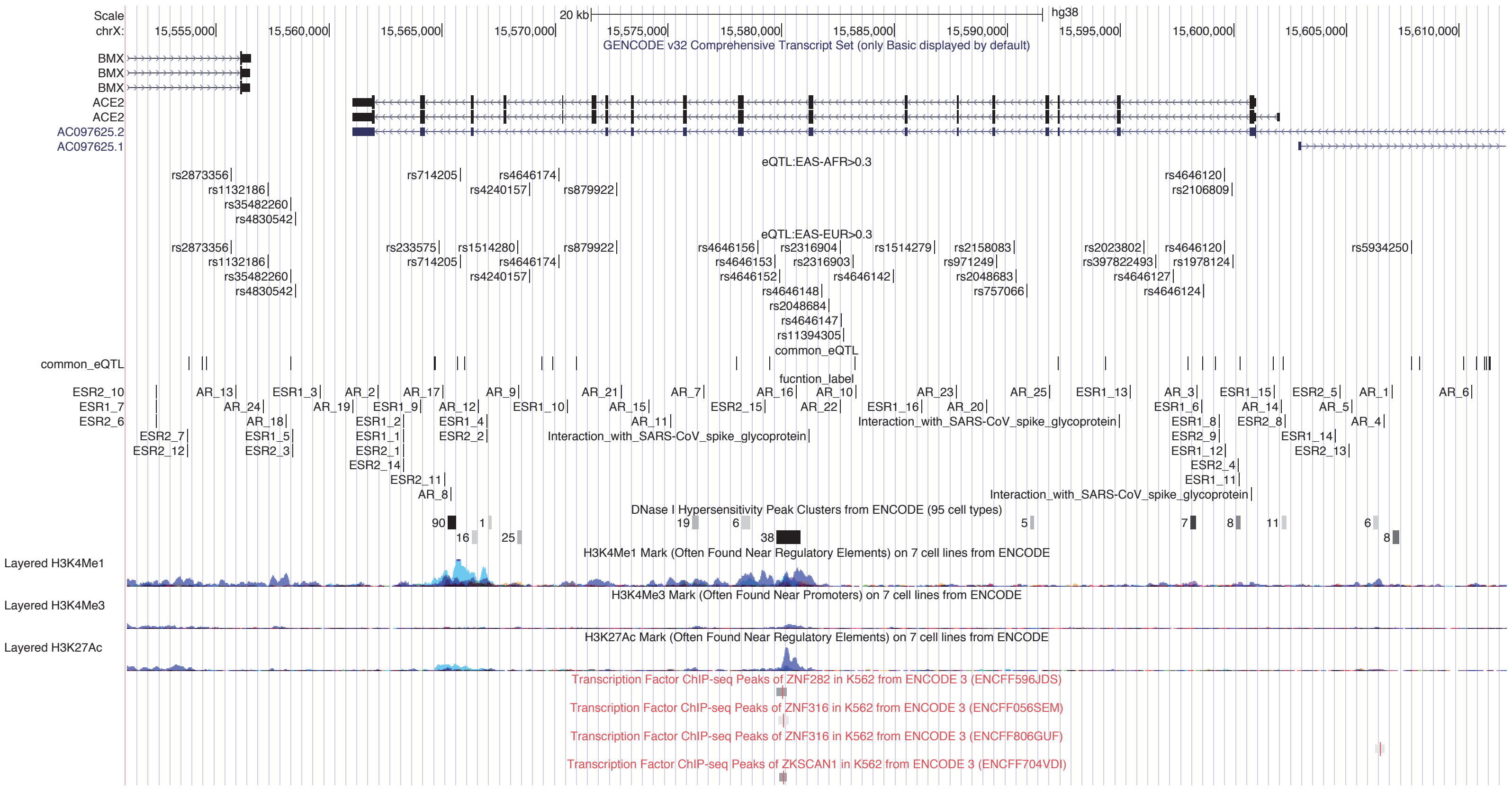

Supplementary Figure 5

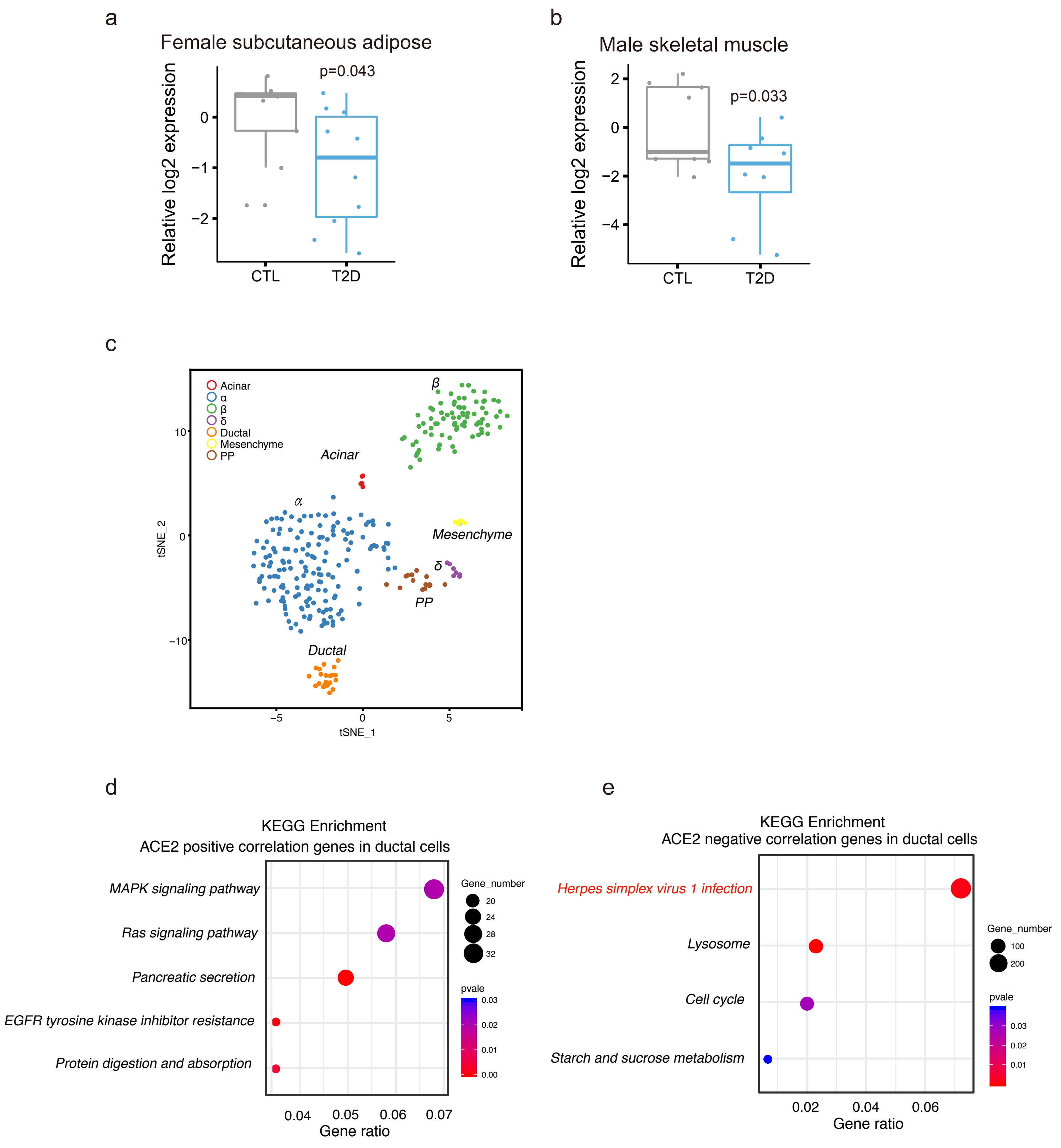

Supplement: Supplementary file 1 — Fig S1‐S5 [file ACEL-19-e13168-s001.pdf]
